# A method in non-destructive testing for lead shielding exceeding 25 *kg/m*^2^ using ^18^F

**DOI:** 10.1007/s13246-025-01524-2

**Published:** 2025-03-11

**Authors:** Jeremy Berrell, Deborah Carrick, Jason Tse, Elaine Ryan

**Affiliations:** 1 Biomedical Technology Services, Queensland Health, Brisbane, Australia; 2 Queensland University of Technology, Brisbane, Qld, 4000 Australia; 3 Forensic and Scientific Services, Coopers Plains, Brisbane, Qld, 4108 Australia; 4 Gold Coast University Hospital, Parkwood, Qld, Gold Coast, 4215 Australia; 5 Prince Charles Hospital, Chermside, Qld, 4032 Australia; 6 Queensland Children’s Hospital, South Brisbane, Qld, 4101 Australia

**Keywords:** Non-destructive testing, Positron emission tomography, PET shielding, Monte Carlo, Fluorine-18, Lead shielding

## Abstract

Non-Destructive Testing (NDT) is a commonly used technique for barrier verification within radiation protection, ensuring compliance with national standards and state regulations. There are currently limited published methods in NDT for lead shielding above 25 *kg/m*^2^, and thus this research aimed to develop a reproducible method to aid in *‘in the field’* NDT for lead barriers exceeding 25 *kg/m*^2^ using a Fluorine-18 (^18^F) source. Due to the fast decay of ^18^F, the data generated within this research was compiled from Monte Carlo (MC) simulations using the PENELOPE engine, and the PENGEOM geometry system to model the proposed empirical setup. The model predicted the Transmission Factor (TF) through area densities (thickness) of lead attenuators up to 302.7 *kg/m*^2^, with results validated by empirical measurements using a Source-to-Detector Distance (SDD) of 38.1 ± 0.05 *cm* and 52.7 ± 0.05 *cm*. However, the study was limited by the chosen activity of ^18^F at approximately 180 *MBq*, where the simulated TF curves demonstrated correspondence of data up to and including area densities of 162.4 *kg/m*^2^ using a 38.1 ± 0.05 *cm* SDD, and 138.0 *kg/m*^2^ with a 52.7 ± 0.05 *cm* SDD. Beyond these thicknesses, the empirical transmission curves deviated from simulated curves due to measurable transmissions becoming significantly reduced. This research demonstrated that using SDDs above 23 *cm* would provide sufficient *near* narrow beam conditions with the proposed experimental configuration for *in-the-field* NDT. The research aimed to develop an equation and method for NDT using a ^18^F source for lead barriers greater than 25 *kg/m*^2^, with transmission data to be made available upon request to the author.

## Introduction

Ensuring the safe use of radiation in facilities, such as hospitals using radiation for medical imaging or treatment, demands meticulous adherence to shielding regulations to meet legislative standards [1–3]. Typically, a radiation shielding plan is designed before any construction to ascertain the required shielding across all barriers within the facility. This is based on equipment/source workloads; the facility design and occupancy factors; the type of radiation equipment used; the specific procedures performed, and the construction materials of the facility itself. Furthermore, it is considered best practice, and in some jurisdictions obligatory, to validate the installed barrier materials and their area density (thickness) post-construction [1–4].

Non-Destructive Testing (NDT) is a method of analysis used for determining the composition and properties of materials, whilst rendering the product undamaged and intact [5, 6]. This can be performed by several techniques such as penetrating radar; laser scanners; infrared; ultrasonic and acoustic emission; along with others [6, 7]. In radiation shielding, this is typically performed by placing a known radioactive source against the barrier to ensure consistent transmission, with a detector positioned on the opposite side, typically at an air gap of 30 cm to minimise scatter contamination. This setup enables measurement of the material’s transmission properties and determination of its area density (thickness).

This can be done empirically, through measurements, or theoretically with a Monte Carlo (MC) model of the measurement set-up. Both can then establish the calibration for the system, ready for *in-field* verification of barriers.

MC simulations have proven to be an effective and efficient tool for the reduction of challenges associated with conducting empirical measurements when establishing the calibration of the measurement system [8–11]. It is a requirement to accurately model all elements of the system used for the barrier measurements, including the isotope container and radiation detector to be used within the MC simulations, with data to be validated either empirically or against published literature.

Once a validated MC model is established then this can be applied to different exposure situations, such as different barrier materials or radioisotopes, without repeating the empirical measurements for all potential scenarios, whilst minimising unnecessary radiation risks to the exposer. Empirical measurements are the gold standard, but if set up correctly, both methods can be used to calibrate the measurement system. Currently, NDT verification is routinely performed using a ^57^Co source, which is good for standard barriers up to 25 *kg/m*^2^ [5].

The motivation for this work was to expand on current experimentally collected ^57^Co NDT data by Lee and Schick [5], due to some medical facilities requiring assessment of lead barriers above 25 *kg/m*^2^. These being facilities that carry out nuclear medicine procedures that utilise high-energy photons, including positron emitter ^18^F, as well as therapeutic isotopes like ^131^I.

This project aims to establish a calibration of an NDT measurement system (source and detector) using a theoretical MC model, using ^18^F. The measurements will also be performed empirically using the measurement configuration design and the results from both methods will be compared. This will lead to a model that will be versatile, as it can be applied to other isotopes and barrier materials, without the empirical calibrations having to be performed, saving time, money, and exposure of workers. It adds to our existing knowledge as it applies to the measurement of lead barriers exceeding 25 *kg/m*^2^, which is a limitation of current methods. The work will also identify any uncertainties associated with the method including finding the optimum measurement distances to be used and assess the maximum practical thickness of lead that can be measured using an ^18^F source.

### Background and previous literature

The Transmission Factor (TF) is vital for NDT as it quantifies the attenuation of radiation through materials or barriers. For accurate NDT, narrow beam geometries are preferred because they reduce scatter, allowing the TF to attest only the primary beam’s attenuation [12]. This creates a straightforward, exponential relationship between TF and barrier thickness, leading to precise calculations of barrier effectiveness. In contrast, broad beam geometries introduce scatter build-up, complicating the TF and necessitating additional corrections. Thus, narrow beam geometries provide a more reliable and accurate method for evaluating the integrity and performance of radiation barriers in NDT applications.

When determining the TF, it is important to distinguish whether the observed transmissions are from broad or narrow beam geometries. Broad beam geometry, characterised by an uncollimated radiation source, results in primary beam attenuation accompanied by scatter build-up. This configuration mirrors realistic scenarios encountered within medical facilities, where barriers shield against uncollimated scattered radiation and/or isotropic emissions. Therefore, utilising broad beam TF is *necessary* when designing shielding requirements, as they offer a more realistic representation of radiation exposure.

For NDT purposes, narrow beam conditions with collimated sources are preferable because they reduce the need for corrections from the excess scatter, allowing the transmission to be described solely by the attenuation of the primary beam [12]. This simplification results in the TF being a single exponential function of barrier thickness, facilitating more accurate calculations of TFs (no scatter) and ensuring precise determination of barrier properties.

This can be conceptualised by considering an infinitely narrow-collimated beam detected at a point infinitely far away, making the scatter contribution effectively zero as it reaches the detector, therefore any reduction in beam intensity is due to attenuation through material from the direct beam [13]. If the material is known, then characteristics such as thickness, can be derived.

Whilst broad beam methods are abundant within literature, they are primarily suited for shielding design calculations and may not directly apply to NDT without corrections. There is extensive narrow beam data available, including mass attenuation coefficients for various materials across different energy spectra [14]. However, this data does not accurately match how NDT is performed in a practical application. There is a blend between creating *‘near’* narrow beam geometries, with a reduced element of build-up scatter.

This makes developing a method that ensures the safety of the operator performing the NDT whilst creating *near* narrow beam geometries challenging. This challenge arises from the need to handle a radioactive source effectively, ensuring adequate protection while achieving the desired narrow beam conditions for accurate NDT measurements. The current availability of methods is limited, especially for lead shielding above 25 *kg/m*^2^.

As the MC modelling of the proposed ^18^F source does not account for time decay, the empirical data would need to be time-adjusted to match simulation data.

1 $$\:{I}_{adj}=\frac{I}{\begin{array}{c}{e}^{-\lambda\:t}\\\:\:\end{array}};$$

where *I*_*adj*_ is the adjusted intensity reading, *I* is the recorded intensity at time *t* after the initial unattenuated intensity *I*_0_ reading, using the decay constant *λ* = 6586.2 ± 1.08 *s*^− 1^ [15].

The adjusted reading is used to calculate the TF,

2 $$\:TF=\frac{{(I}_{adj}-{I}_{B})}{\left({I}_{0}-{I}_{B}\right)\times\:IS{L}_{cor}};$$

where *I*_*B*_ is the ambient background reading per location/occasion, *I*_*B*0_ is the background reading taken at the location of *I*_0_, and *ISL*_*cor*_ is the Inverse Square Law (ISL) correction if there is a change in SDD between their respective readings,

3 $$\:IS{L}_{cor}={\left(\frac{SD{D}_{0}}{SDD}\right)}^{2};$$

where *SDD*_*0*_ is the SDD at measurement *I*_*0*_, and *SDD* is at measurement *I*_*adj*_.

The TF curve equation used for determining the thickness of lead attenuators is expressed in the form.

4 $$T F=\ C e^{-\mu x}$$

where *µ* is the linear attenuation coefficient, *x* is the thickness of the attenuator, and *C* = 1 is the y-intercept for incident TF at *x* = 0, i.e. *TF* = 1 with no attenuation.

The current research has been modelled from the NDT work outlined by Lee and Schick [5]. They identify that there is a lack of published data on transmission properties of radioisotopes utilised in NDT for shielding materials in medical imaging facilities [5]. To address this, they measured the attenuation of a ^57^Co source through lead attenuators in thicknesses commercially available for X-ray shielding, i.e. 5 *kg/m*^2^ increments [5]. Their results provided a set of equations that can be used in NDT assessments to predict the transmission of radiation through shielding materials, along with the associated uncertainties using a ^57^Co source for lead barriers up to 25 *kg/m*^2^ [5].

Madsen et al. provide TFs for radionuclides commonly used in PET, focusing primarily on the 511 *keV* annihilation photons of ^18^F [16]. They present TFs for lead, concrete, and iron barriers using an experimental setup based on designs by Archer et al., which employs broad beam configurations [17]. While this work is sufficient for shielding design, assumptions and corrective factors would need to be applied for use in NDT applications.

## Materials and methods

The current work aimed to create a method in NDT for lead above 25 *kg/m*^2^ however, to establish a reasonable experimental endpoint, a preliminary survey was conducted through Queensland Health’s physics departments to determine the maximum installation thicknesses throughout. A consensus was that most PET departments ranged between 25 *kg/m*^2^ in the PET/CT imaging rooms up to 100 *kg/m*^2^ for a standard patient uptake room, with maximum installations as high as 170 *kg/m*^2^. This project simulated and empirically measured up to 302.7 *kg/m*^2^, for future expansion.

### Measurement configuration

#### Equipment

The measurement configuration consisted of a Ludlum Model 44 − 2 Gamma Scintillator (Ludlum Measurements Inc., Texas, USA) used for the detection process, in conjunction with a Ludlum Model 2241-2 Digital Scaler-Ratemeter [18, 19]; a 20 *mL* vial ^18^F (in the form of 18F-FDG); a MEDRAD Intego- Blue Vial Shield (Medrad Inc., California, USA) tungsten containment pot, with conical collimating lid with 4 *mm* aperture necessary for creating *‘near’* narrow beam conditions [20]; as well as 60 × 60 *mm*^2^ lead attenuating plates of varying indicated thicknesses.

The lead plates used had a given indicated thickness per plate, however, as a secondary measure each plate was weighed 10 times to derive an area density based on their dimensions. The uncertainty in lead was attained from the standard deviation in their weight with propagation against the uncertainty in their given values.

#### Setup

The following measurement configuration is used throughout both the MC modelling and empirical validation stages of the current work.

**Fig. 1:**
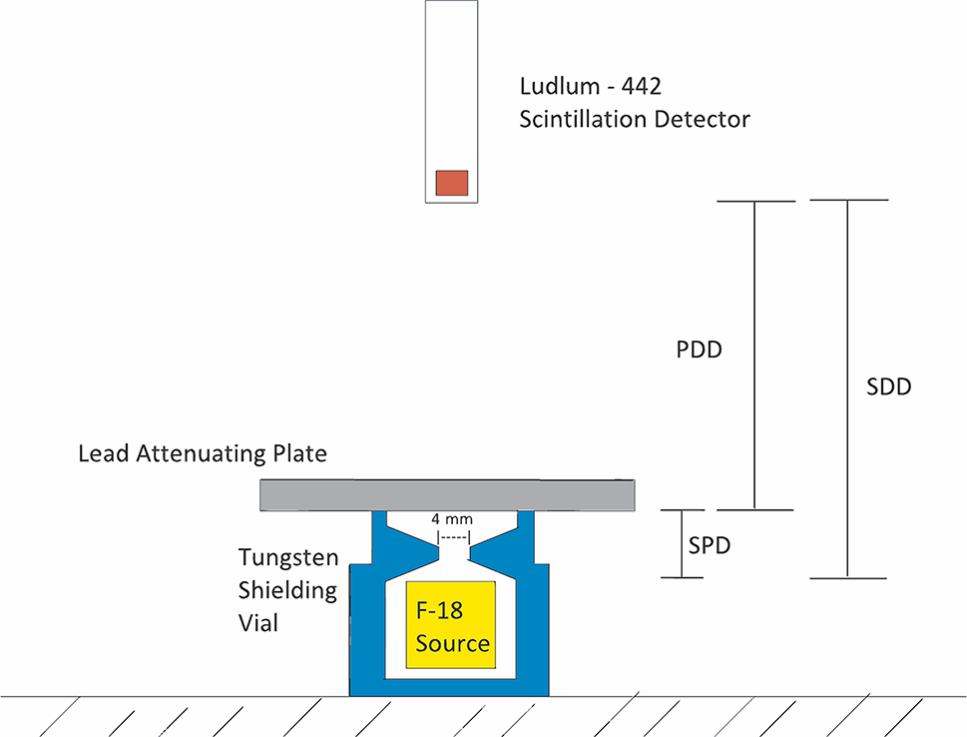
The setup consists of a ’Ludlum Model 44 − 2 Gamma Scintillator’ with internal NaI detection crystal, a 20 *ml* ^18^F vial, a ’Medrad Intego- Blue Vial Shield’ tungsten containment pot, and 60 × 60*mm*^2^ lead attenuating plates of varying thicknesses

Below in Fig. 1 is a schematic of the configuration, including the collimated Blue Vial Shield tungsten lead pot with 4 *mm* aperture, Ludlum Model 44 − 2 Gamma Scintillator with NaI detection crystal, a 20 *ml* ^18^F source, and lead attenuation plates. The design includes the Source-to-Detector Distance (SDD), Pot to Detector Distance (PDD), and Source-to-Pot-exit Distance (SPD) of 3 *cm*.

### Monte Carlo simulation

The measurement setup outlined in Fig. 1 was modelled using the Pengeom quadric geometry system, with materials sourced using the PENELOPE Pendbase library of elements and compositions [21–23].

The simulation was run in a vacuum state to reduce unnecessary interactions, reducing the computational workload.

The model of the Ludlum 44 − 2 used dimensions outlined within the specification manual [18], which consisted of aluminium housing; a NaI scintillation crystal abut a CsSb photocathode cylinder to emulate the Photo-Multiplier Tube (PMT); With internal space between external housing and NaI crystal filled with air rather than the foam specified within the manual [18]. However, due to the simulation running in a vacuum, it was assumed that this exchange of foam to air is equivalent and/or negligible with the use of high-energy 511 *keV* photons.

The lead attenuation plates were modelled using a rectangular prism of fixed lengths 60 × 60 *mm*^2^, with varying thicknesses of attenuation to match available lead, with the lead attenuator abut the Blue Vial Shield tungsten containment pot [20]. The ^18^F source was modelled as a monoenergetic 511 *keV* photon emitter from a cylindrical vial of water of dimensions 35 *mm* length and 13.5 *mm* radius, to emulate a full vial, with vial housing ignored for simulation.

**Fig. 2:**
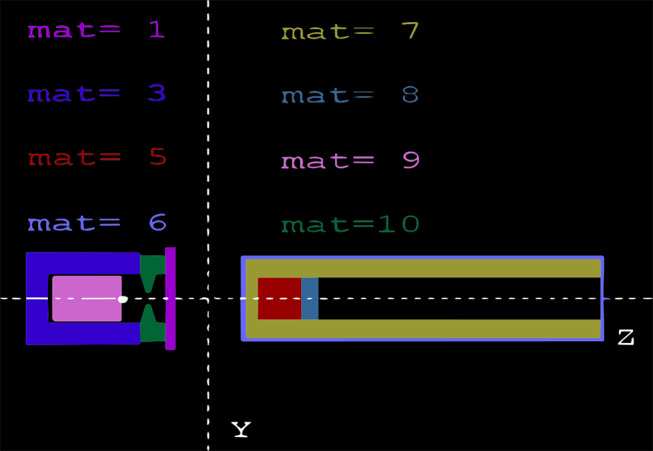
Mat 1 - Lead attenuation plate (fuchsia); Mat 3 - Tungsten vial body (dark blue); Mat 5 NaI crystal (red); Mat 6- Aluminium housing of Ludlum 44 − 2 detector (light blue); Mat 7 - Air insert surrounding crystal and inside aluminium housing of Ludlum 44 − 2 detector (khaki/pale green); Mat 8 - Photocathode abut scintillation crystal from the PMT (pale aqua); Mat 9–18 F source (pink); Mat 10 - Tungsten vial lid (green)

Noting that the dimensions of Blue Vial Shield and vial were empirically measured with all modelled material densities sourced using the Pendbase Pengeom data bank [21]. The objects and arrangement for the simulation can be seen in 2D view in Fig. 2, using PENELOPE’s gview2d [21].

The constant parameters throughout all simulations were as follows: each simulation ran using the variance reduction techniques particle splitting, with 1000 splits per history within the NaI crystal, as well as Russian roulette, using a survival probability of 0.1% within the body of the tungsten vial (excluding the lid) [24]; an Azimuthal angle of 360^◦^ and polar angle of 90^◦^, with direction vector *z* = 1^1^; all simulated materials were assigned a photon cut-off energy of 1 *keV*, except the detector crystal which was assigned a photon cut-off energy of 80 *keV*. This was chosen to bias the counts of photons within the detector crystal to reduce uncertainty. Noting that only 511 *keV* photons were simulated, rather than the positron annihilation process to reduce computational workload. All simulations ran for varying histories which will be discussed explicitly at each stage.

The first simulation aimed to determine the optimal SDD for achieving near-narrow beam conditions in the measurement setup. SDD was varied from 3 cm to 53 cm across lead barriers of 24.6 *kg/m*^2^ and 138.0 *kg/m*^2^ using MC simulations. Energy Deposition (ED) in the detector was recorded for each SDD configuration. The analysis involved applying an ISL-predicted ED *ED*_*ISL*_ to the simulated ED *ED*_*SIM*_ values using Eq. (4), to assess scatter contribution at each SDD. Using the Simulated ED energy at the farthest SDD as the *reference ED* and *reference distance*, the predicted *ED*_*ISL*_ was determined for the next closest *distance* SDD point simulated. This was repeated sequentially toward the detector to determine the SDD where scatter detection had been minimised.

5 $$\:E{D}_{ISL}=\frac{reference\:ED}{{\left(distance/reference\:distance\right)}^{2}}$$

From the results of the SDD simulations, subsequent simulations were conducted using SDDs above 30 *cm* to generate data for constructing TF curves necessary for area density extrapolation. Each simulation ran 2 × 10^9^ histories within the range of 33 *cm* to 73 cm SDD, with 5 *cm* increments. ED was recorded within the NaI detection crystal for each thickness independently.

The NDT equation developed from the simulation data was formed using the MATLAB (Mathworks, Massachusetts, US) curve fitting toolbox, and MATLAB R2023b for all graphs generated throughout this report [25].

### Empirical verification

The empirical reference data was collected in two independent experiments: the first using an SDD of 38.1 ± 0.05 *cm* using a ^18^F source with activity ≈ 185 *MBq* at the time of first reading, and the second using 52.7 ± 0.05 *cm* SDD with an activity of ≈ 180 *MBq*. As ^18^F has a half-life of ≈ 110 *min,* both experiments were conducted in less than 2 h per experiment.

The Ludlum Model 44 − 2 Gamma Scintillator was used for detection, with a Ludlum Model 2241-2 Digital Scaler-Ratemeter [18, 19]. The ratemeter was set to take readings using a variable response and slow integration [19]. Meter readings were given in *µSV/hr* with ± 0.5 *µSv/hr* for readings above 5 *µSv/hr* and ± 0.0005*µSv/hr* for readings less than 5 *µSv/hr*, from meter display limitations. Readings were taken every 1 min to maximise the count integration, with readings taken twice per thickness in ascending order, and twice in descending order to maximise readings with consideration for the decay of the ^18^F source whilst minimising uncertainty. The PDD was measured using a Leica Disto™ A8 laser meter (Hexagon, Stockholm, SWEDEN), with uncertainty in readings of ± 0.5 *mm* [26].

The detector and the source were set on independent tables with an air gap between them to reduce additional scatter, at a height of 1.050 ± 0.0005 *m* to the centre of the source and detector for both experiments, with SDD previously discussed. A 20 *ml* ^18^F-FDG vial was used in both experiments to its maximum fill line to ensure reproducibility and uniformity of the source.

All graphs generated throughout this report were created using MATLAB R2023b [25].

## Results

### Monte Carlo simulation

**Fig. 3:**
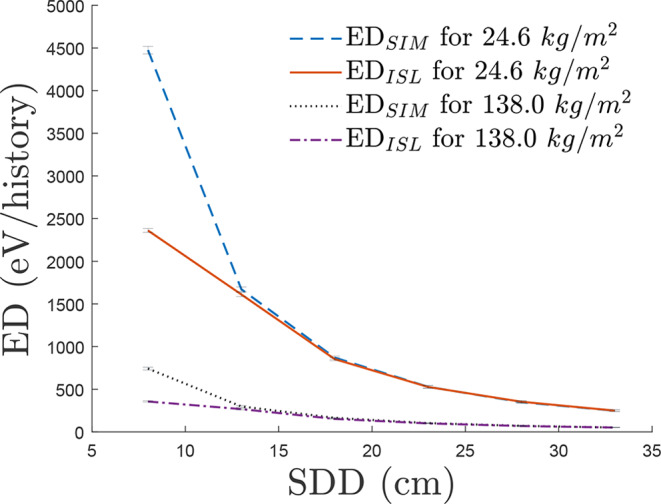
ED_*SIM*_ is the simulated ED data in NaI plotted against a predicted curve ED_*ISL*_ using ISL corrections at each SDD for 24.6*kg/m*^2^ and 138.0 *kg/m*^2^ to establish an SDD that minimises the scatter contribution, showing an alignment in each simulation at ≈ 23 *cm* SDD

The first series of simulations was used to establish a suitable SDD to ensure that any scatter contribution due to the finite size of the aperture of the tungsten container was neglected in the measurements, to achieve near-narrow beam conditions. Using Eq. (4), each neighbouring ED value was analysed and compared to the tallied ED value given, as seen in Fig. 3, with SDD’s 8 *cm* through to 33 *cm* displayed for clarity.

This demonstrated for both simulations, the ED_*SIM*_ data was disproportionate to the ED_*ISL*_ at SDDs below 23 *cm*. With ED_*ISL*_ values and ED_*SIM*_ values corresponding within ± 3% for 138.0 *kg/m*^2^ and ± 0.3% for 24.6 *kg/m*^2^. The simulations were run up to 53 *cm* SDD, with negligible variation between ED_*SIM*_ and corresponding ED_*ISL*_ calculations at higher SDD.

**Fig. 4:**
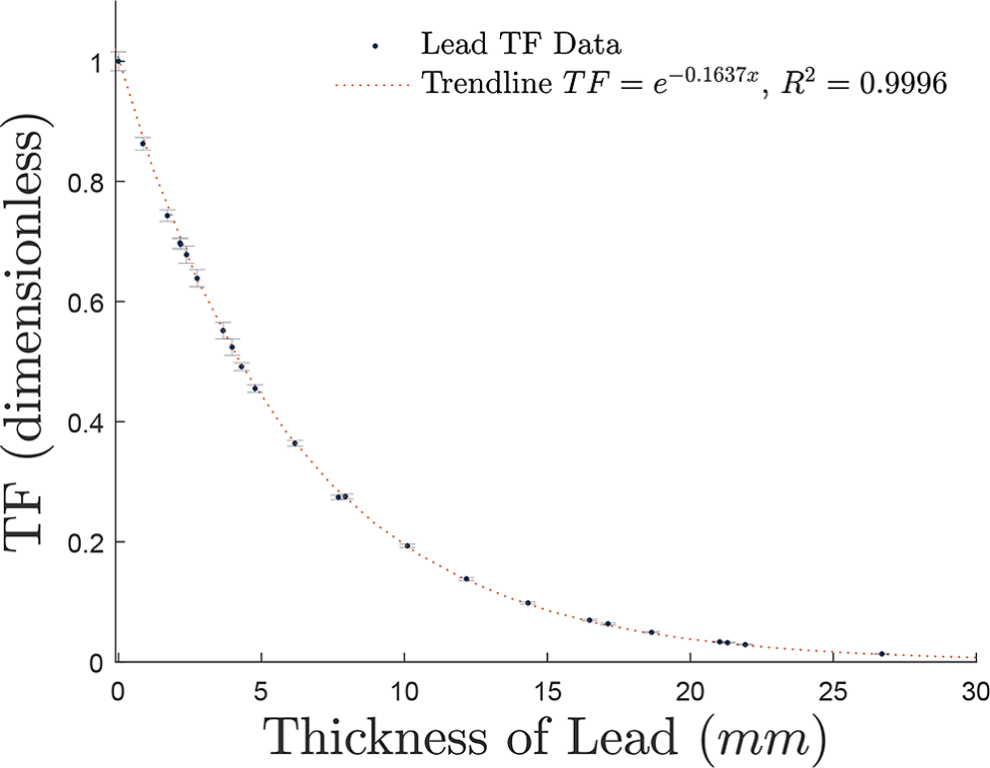
The TF curve for lead was obtained by averaging the curves of all simulated SDDs above 33 *cm* using 2 × 10^9^ histories, with uncertainties propagated into error bars. The resulting fit curve is expressed as *TF*_*lead*_ = *e*^−0.1637*x*^, with an *R*^2^ value of 0.9996

For the simulation of lead, the mean of the TF and varying SDD simulation data was taken and displayed in Fig. 4, along with its best-fit equation to be used in NDT.

As the SDD used were all above 33 *cm* the corresponding TF curves fit over the same line with negligible variation, therefore the mean of the simulations was taken to create the curve in Fig. 4 in the form of Eq. (3) with *TF* = *e*^− 0.1637*x*^ and *R*^2^ = 0.9996. Taking the inverse function and rearranging for *x*, the equation for the thickness *x* (*mm*) becomes

6 $$\:{x}_{lead}\left(mm\right)=\frac{\text{ln}\left(1/TF\right)}{0.1637}\:mm.$$

### Empirical verification

**Fig. 5:**
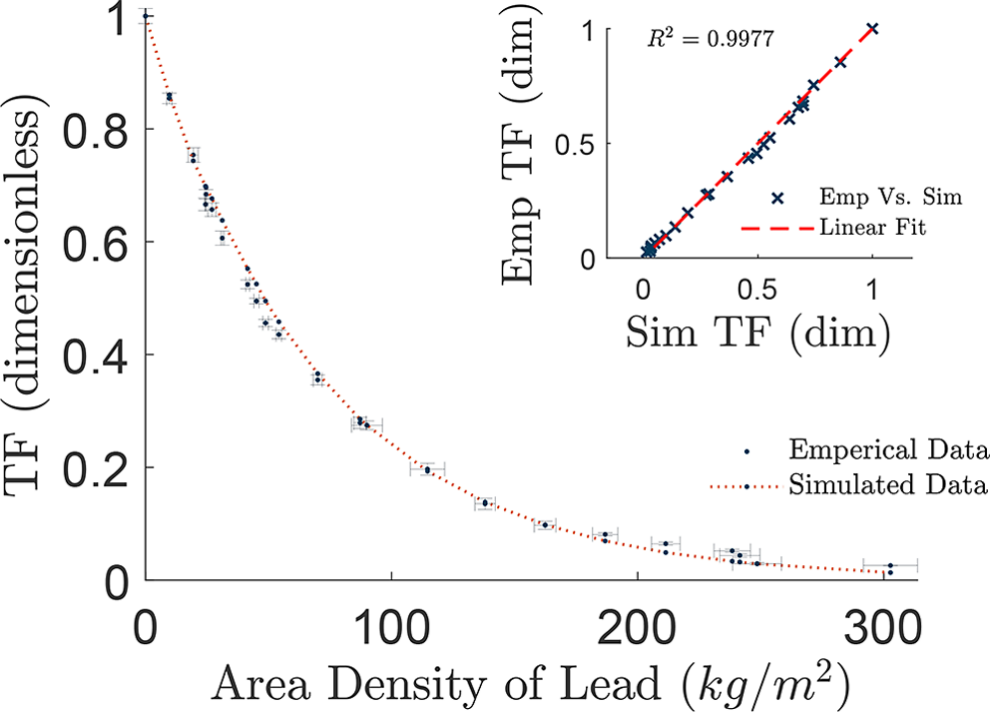
Empirically (Emp) measured data against MC Simulated (Sim) results using a 38.1 ± 0.05 *cm* SDD. The embedded graph in the upper right quadrant shows the linear correlation of empirical measurements vs. simulated data, with a correlation coefficient of *R*^2^ = 0.9977

The physical measurements were acquired in two separate experiments. The first ran using a 38.1 ± 0.05 *cm* SDD and collated with simulation data collected using 38.1 ± 0.05 *cm* SDD as seen in Fig. 5. The experiment ran with a background reading of *I*_*B*_ = 0.197 ± 0.006 *µSV/hr*, with unattenuated reading of *I*_0_ = 66 ± 0.5 *µSv/hr* and lowest attenuated reading of *I* = 1.385 ± 0.0005 *µSv/hr*.

**Fig. 6:**
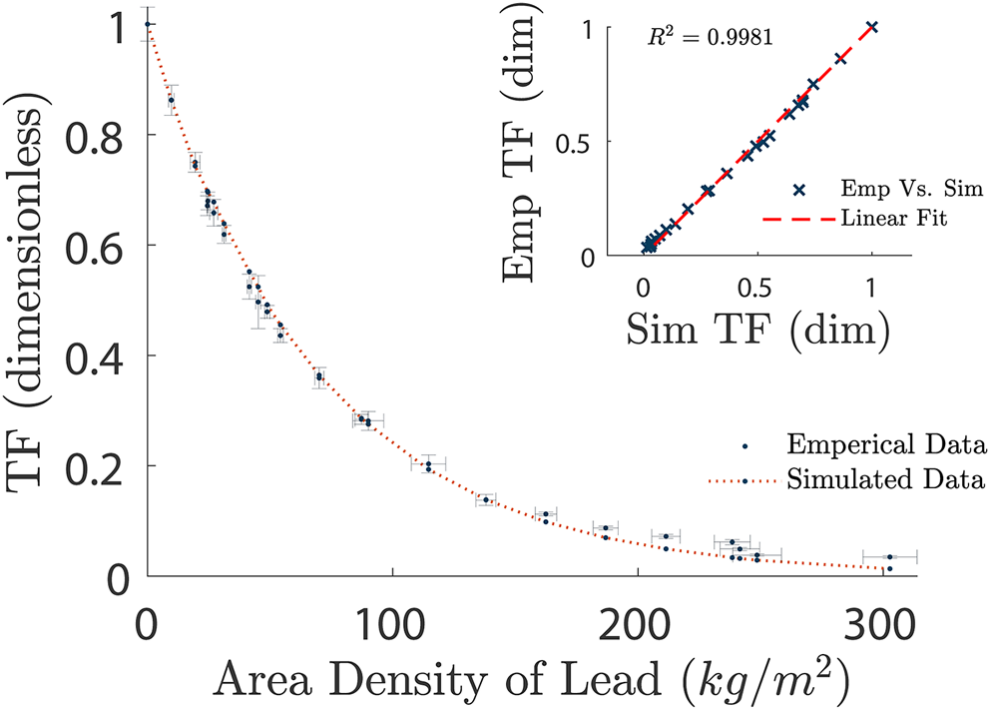
Empirically (Emp) measured data against MC Simulated (Sim) results using a 52.7 ± 0.05 *cm* SDD. The embedded graph in the upper right quadrant shows the linear correlation of empirical measurements vs. simulated data, with a correlation coefficient of *R*^2^ = 0.9981

The empirical measurements were compared against the simulated data to attain their linear correlation, giving a correlation coefficient of *R*^2^ = 0.9977. The simulated data correlated well at low attenuation within predicted error margins, however, after approximately 162.4 ± 4.4 *kg/m*^2^ there is a deviation in measured values from the simulation displaying a higher recorded TF than given by the simulation. It can similarly be seen in Fig. 6, where the empirical data using 52.7 ± 0.01 *cm* SDD with simulated data using 52.7 ± 0.05 *cm* SDD. The experiment ran with a background reading of *I*_*B*_ = 0.186 ± 0.004 *µSV/hr*, with unattenuated reading of *I*_0_ = 46 ± 0.5 *µSv/hr* and lowest attenuated reading of *I* = 1.212 ± 0.0005 *µSv/hr*.

**Fig. 7:**
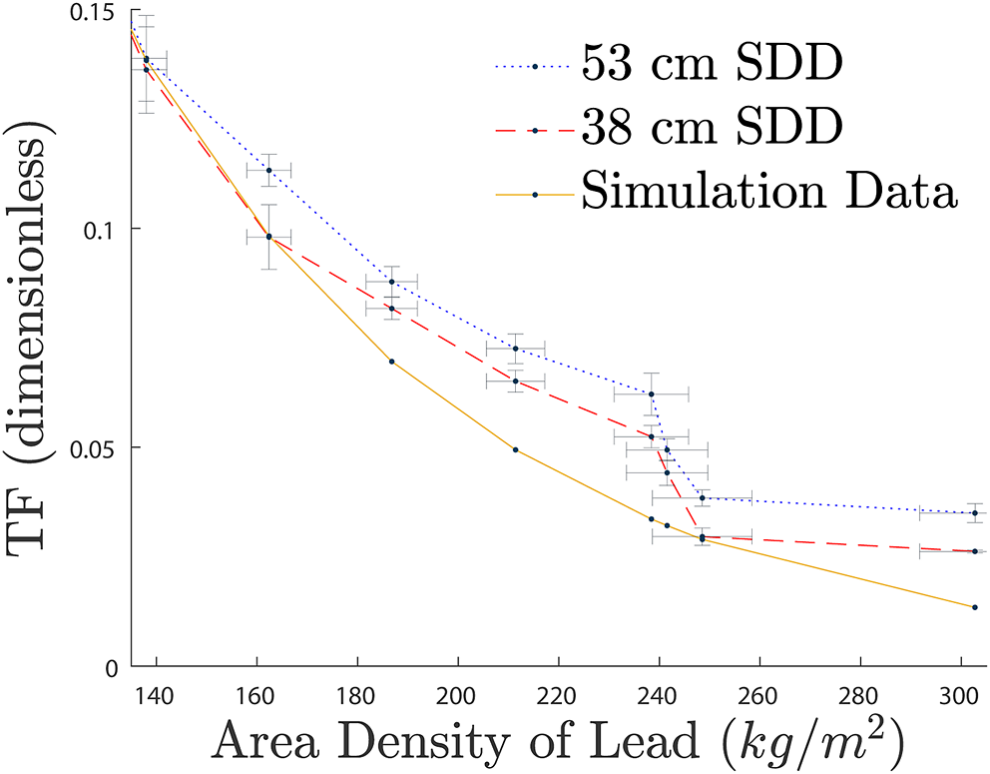
Comparison of TF curves for the simulation data with the measured data at 38.1 ± 0.05 *cm* and 52.7 ± 0.05 *cm*, showing a deviation from the predicted TF for both, with a greater deviation at higher SDD

The data demonstrated a similar correlation up to attenuators of approximately 138 kg/m^2^, beyond which there was an increase in TF compared to the predicted simulated data. With a minor increase in the deviation from TF for 52.7 ± 0.05 *cm* compared to 38.1 ± 0.05 *cm* SDD as seen in Fig. 7, with a focus on attenuation curves between 135.0 *kg/m*^2^ to 302.7 *kg/m*^2^. Similarly, the data sets can be seen to have a high level of correlation, with empirical against simulation data giving a linear correlation of *R*^2^ = 0.9981.

## Discussion

The concept of this research is one based on practicality, with particular importance in the tungsten containment pot having a conical collimating cap with 4 *mm* aperture, necessary to achieve near-narrow beam geometry. The Blue Vial Shield is not a custom piece of engineering specifically crafted for this project; therefore it can be commercially purchased by any facility that can acquire ^18^F looking to perform NDT, not limited to PET facilities alone [20]. The second practicality is in the choice of the ^18^F source, due to its abundance in almost any PET facility (Typically in the form of ^18^F-FDG). The ^18^F-FDG can be drawn as required to a specified activity based on the level of NDT barrier thicknesses. The activities used were chosen as a balanced compromise, aiming to minimise uncertainties in the measurement process with higher activity levels, whilst ensuring radiation safety for handling the source to minimise exposure and account for the decay of ^18^F.

A key focus of this research was to explore and determine a suitable SDD that would significantly reduce scatter for the proposed experimental design, creating a close-to-narrow beam geometry. It is important to note that the conditions created are not truly narrow beam as a persistent minimal degree of scatter contribution is present.

The simulations used in Fig. 3 were run up to and including 103 *cm* SDD, displaying a degree of scatter contribution due to the finite size of the aperture.

However, as NDT is performed from *in the field* measurements, choosing a significantly large SDD will bring challenges (e.g., low counts, errors with positioning, and higher activity requirements). The alignment shown in Fig. 3, shows that at an SDD as small as 23 *cm*, the *near* narrow beam conditions are adequately defined within ± 3% for 138.0 *kg/m*^*2*^ and ± 0.3% for 24.6 *kg/m*^*2*^. Based on this result, the data generated and empirical validation was decided to be conducted above 30 *cm* SDD, to minimise scatter, whilst retaining a suitable SDD for practical NDT application.

The simulation curve presented in Fig. 4 is the mean of the simulated data for all SDDs above 30 *cm*. This approach was deemed appropriate as all subsequent trendlines of SDDs over 30 *cm* followed the same exponential fitting curve when overlaid, further proving the minimal detection of scatter.

The simulation data was generated in millimetres of lead due to constraints in PENELOPE, and was converted to area density (using a density of 11, 350 *kg/m*^*3*^, as per the Pengeom Pendbase materials library) to match the empirical values indicated in Figs. 5, 6 and 7. This allowed the simulation results to correspond accurately with the respective SDDs.

For the empirical validation, the experiment was conducted twice with a week intermission, using a 38.1 ± 0.05 *cm* SDD and 52.7 ± 0.05 *cm* SDD. This was to eliminate any chance of external biasing, such as fluctuations in background radiation, or possible contaminants from the nearby PET facilities. This approach was intended to confirm that the empirical data matched the simulation on separate occasions using different SDDs.

The comparison of these data sets showed significant correlation up to and including 162.4 ± 4.4 *kg/m*^*2*^ using 38.1 ± 0.05 *cm* SDD, and as far as 138.0 ± 4.1 *kg/m*^*2*^ using a 52.7 ± 0.05 *cm* SDD, with an increase of TF above these area densities as seen in Fig. 7. There are several reasons this could be accredited to, such as the compiled uncertainty in the lead plates as multiple plates were needed to attain the larger thicknesses of lead; limitations within the detector itself; room and bench scatter, where the simulated model has an absence of peripheral objects; or the inability of the source to penetrate at the chosen level of activity as the readings became closer to background. Noting a larger uncertainty in measurements with lower counts for high attenuation.

The linear correlation of Empirical Vs. Simulated TF datapoints, showed excellent correspondence, with 38 *cm* SDD having a correlation coefficient of R^2^ = 0.9977 in Fig. 5, and 53 *cm* SDD having a coefficient of R^2^ = 0.9981 in Fig. 6. Even with the deviation at low TF, the linear correspondence suggests the MC model accurately depicted the empirical design.

Interestingly, the TF curve for 52.7 ± 0.05 *cm* SDD has a greater deviation from the simulated line than the curve for 38.1 ± 0.05 *cm*. This is most likely due to the reduction of intensity from ISL from the chosen activity, giving the 38.1 ± 0.05 *cm* curve a better fit at high attenuation. As both empirical data acquisitions were conducted using a source of approximately 180 *MBq*, should a source with a higher activity be used, it is assumed a closer fit to the simulated data would be achieved, however further experimentation is needed to confirm this.

### Limitations and considerations

The limitations of this study were due to time and resource constraints. The lead borrowed was from the Queensland University of Technology, although it is intended for experimental purposes and assumed to be of a high purity, true specifications could not be confirmed adding an additional degree of uncertainty. The time in which the lead plates could be borrowed was also limited which restricted the number of empirical validation experiments. Consequently, a source activity of approximately 180 *MBq* was chosen as a balance between primary beam penetration and operator safety, leading to discrepancies at high attenuation for area densities above 162.4 ± 4.4 *kg/m*^2^, with the belief that a greater source activity would yield more correlated results at higher attenuation.

The key understanding of these discrepancies is that the equation developed in this research aims to support practical applications in NDT. Uncertainties in the transmission data are expected to be less pronounced comparatively to uncertainties in the physical measurement process, such as variations in SDD, and source and detector positioning on either side of a barrier. In Australia, barrier installations typically follow increments of 5 *kg/m*^2^ due to manufacturing constraints, leading to significant rounding of results.

This research has also generated similar attenuation curves for gypsum (2,320 *kg/m*^3^) and concrete (2,300 *kg/m*^3^), with future works to perform similar experiments to validate their accuracy, with any transmission data to be made available upon request to the author.

## Conclusion

This research has developed a novel method for conducting NDT by leveraging commonly available equipment found in PET facilities, for practical *‘in the field’* calculations.

The equation *x*_*lead*_* (mm)* = ln*(TF*^*-1*^*)* × 0.1637^−1^, was derived using MC simulations with use of SDDs above 23 *cm*, with empirical data validated up to 162.4 ± 4.4 *kg/m*^2^ using a 38.1 ± 0.05 *cm* SDD, and 138.0 ± 4.1 *kg/m*^2^ with 52.7 ± 0.05 *cm* SDD. Beyond these SDDs, there were noticeable deviations from the simulated predictions. These deviations were likely associated with the higher uncertainty in low dose rate measurements and may be reduced by using an ^18^F source with initial activity greater than 180 *MBq* if accuracy in high attenuating shielding is required. However, further research is needed to validate this assertion.

To conclude, this study developed an equation for *in-the-field* NDT of lead installations above 25 *kg/m*^2^, with empirical validation as high as 162.4 ± 4.4 *kg/m*^2^, covering thicknesses typically found in PET facilities, i.e. in the order of ≈100 *kg/m*^2^. Consequently, there is a degree of tolerance towards measurement errors due to the typical 5 *kg/m*^2^ incremental installations within barriers, enhancing the practicality and robustness of the method for NDT applications for facilities that use high-energy photons.

## Footnotes

1 This ensured all photons are forward moving in the direction of the detector, with no unnecessary emissions away from the detector.

## References

[1] Australian Radiation Protection and Nuclear Safety Agency (2023) online. https://www.arpansa.gov.au/10.1007/s13246-012-0127-3 22302465

[2] International Atomic Energy Agency (2023) online. https://www.iaea.org/

[3] VALENTIN, J. (ed.) (2007) Annals of the ICRP publication 103. International Commission on Radiological Protection 10.1016/j.icrp.2007.10.003 18082557

[4] Standard for premises–ionising radiation sources (2021) Technical report, Queensland Government (2021). Radiation Safety Standard - Radiation Safety Act 1999

[5] Lee KL, Schick D (2009) Lead attenuation characteristics models. Australasian Phys Eng Sci Med 32(4):212–219. 10.1007/bf03179241 10.1007/BF03179241 20169840

[6] Hanke R, Fuchs T, Uhlmann N (2008) X-ray based methods for non-destructive testing and material characterization. Nuclear instruments and methods in Physics Research Section A: Accelerators, Spectrometers. Detectors Assoc Equip 591(1):14–18. 10.1016/j.nima.2008.03.016

[7] Kot P, Muradov M, Gkantou M, Kamaris GS, Hashim K, Yeboah D (2021) Recent advancements in non-destructive testing techniques for structural health monitoring. Appl Sci 11(6):2750. 10.3390/app11062750

[8] Guerra JG, Rubiano JG, Winter G, Guerra AG, Alonso H, Arnedo MA, Tejera A, Martel P, Bolivar JP (2018) Modeling of a HPGe well detector using PENELOPE for the calculation of full energy peak efficiencies for environmental samples. Nuclear instruments and methods in Physics Research Section A: Accelerators, Spectrometers, detectors and Associated Equipment. 908:206–214. 10.1016/j.nima.2018.08.048

[9] Kroese DP, Brereton T, Taimre T, Botev ZI (2014) Why the Monte Carlo method is so important today. WIRE Comput Stat 6(6):386–392. 10.1002/wics.1314

[10] Zhang F, Zhao X, Zhang J (2020) Simulation of x-ray shielding effect of different materials based on MCNP5. OALib 07(09):1–7. 10.4236/oalib.1106727

[11] Kling A, Nakagawa M, Tavora L, Vaz P (2001) Advanced Monte Carlo for radiation physics, particle transport simulation and applications, 1242

[12] Pernicka F (2007) Dosimetry in diagnostic radiology an international code of practice, 359

[13] Chang DS, Lasley FD, Das IJ, Mendonca MS, Dynlacht JR (2014) Basic Radiotherapy Phys Biology 69–76. 10.1007/978-3-319-06841-17

[14] Seltzer S (1995) Tables of X-Ray Mass Attenuation Coefficients and Mass Energy Absorption Coefficients, NIST Standard Reference Database 126. National Institute of Standards and Technology 10.18434/T4D01F

[15] Unterweger MP, Fitzgerald R (2014) Update of NIST half-life results corrected for ionization chamber source-holder instability. Appl Radiat Isot 87:92–94. 10.1016/j.apradiso.2013.11.017 24321494 10.1016/j.apradiso.2013.11.017 PMC7649977

[16] Madsen MT, Anderson JA, Halama JR, Kleck J, Simpkin DJ, Votaw JR, Wendt RE, Williams LE, Yester MV (2005) AAPM task group 108: PET and PET/CT shielding requirements. Med Phys 33(1):4–15. 10.1118/1.2135911 10.1118/1.2135911 16485403

[17] Archer BR, Thornby JI, Bushong SC (1983) Diagnostic x-ray shielding design based on an empirical model of photon attenuation. Health Phys 44(5):507–517. 10.1097/00004032-198305000-00005 6853171 10.1097/00004032-198305000-00005

[18] Model 44– 2 NaI(Tl (2023) Scintillation Gamma Detector. Ludlum Measurements, Inc. https://ludlums.com/products/all-products/product/model-44-2

[19] Model 2241-2 Digital Scaler-Ratemeter (2023) Ludlum Measurements, Inc. https://ludlums.com/products/all-products/product/model-2241-2

[20] GE Healthcare - Medrad Intego (2023) - Blue Vial Shield Product Overview. online. https://www.radiology.bayer.com/sites/g/files/vrxlpx8576/files/2020-08/86458802.pdf

[21] Salvat F (2014) Penelope-2014: A code system for Monte Carlo simulation of electron and photon transport

[22] Almansa J, Salvat-Pujol F, D´ıaz-London˜o G, Carnicer A, Lallena AM, Salvat F (2021) PENGEOM — a general-purpose geometry package for Monte Carlo simulation of radiation transport in complex material structures (new version announcement). Comput Phys Commun 264:107962. 10.1016/j.cpc.2021.107962

[23] Almansa J, Salvat-Pujol F, D´ıaz-London˜o G, Carnicer A, Lallena AM, Salvat F (2016) PENGEOM —a general-purpose geometry package for Monte Carlo simulation of radiation transport in material systems defined by quadric surfaces. Comput Phys Commun 199:102–113. 10.1016/j.cpc.2015.09.019

[24] Sempau J (2020) Penelope/peneasy user manual 2020

[25] Inc. TM MATLAB Version: 9.14.0 (R2023a). https://www.mathworks.com

[26] Leica Disto™ A8 User Manual (2019) Online. Leica Geosystems https://shop.leicageosystems.com/sites/default/files/2019-03/ leica disto user manual a8 en.pdf

